# Galápagos upwelling driven by localized wind–front interactions

**DOI:** 10.1038/s41598-020-80609-2

**Published:** 2021-01-14

**Authors:** Alexander Forryan, Alberto C. Naveira Garabato, Clément Vic, A. J. George Nurser, Alexander R. Hearn

**Affiliations:** 1 Ocean and Earth Science, University of Southampton, National Oceanography Centre, Southampton, SO14 3ZH UK; 2 Laboratoire d’Océanographie Physique et Spatiale, 29280 Plouzané, Brittany France; 3 National Oceanography Centre, Southampton, SO14 3ZH UK; 4 Galápagos Science Center, Universidad San Francisco de Quito, Quito, 170901 Ecuador

**Keywords:** Marine biology, Physical oceanography

## Abstract

The Galápagos archipelago, rising from the eastern equatorial Pacific Ocean some 900 km off the South American mainland, hosts an iconic and globally significant biological hotspot. The islands are renowned for their unique wealth of endemic species, which inspired Charles Darwin’s theory of evolution and today underpins one of the largest UNESCO World Heritage Sites and Marine Reserves on Earth. The regional ecosystem is sustained by strongly seasonal oceanic upwelling events—upward surges of cool, nutrient-rich deep waters that fuel the growth of the phytoplankton upon which the entire ecosystem thrives. Yet despite its critical life-supporting role, the upwelling’s controlling factors remain undetermined. Here, we use a realistic model of the regional ocean circulation to show that the intensity of upwelling is governed by local northward winds, which generate vigorous submesoscale circulations at upper-ocean fronts to the west of the islands. These submesoscale flows drive upwelling of interior waters into the surface mixed layer. Our findings thus demonstrate that Galápagos upwelling is controlled by highly localized atmosphere–ocean interactions, and call for a focus on these processes in assessing and mitigating the regional ecosystem’s vulnerability to 21st-century climate change.

## Introduction

**Figure 1:**
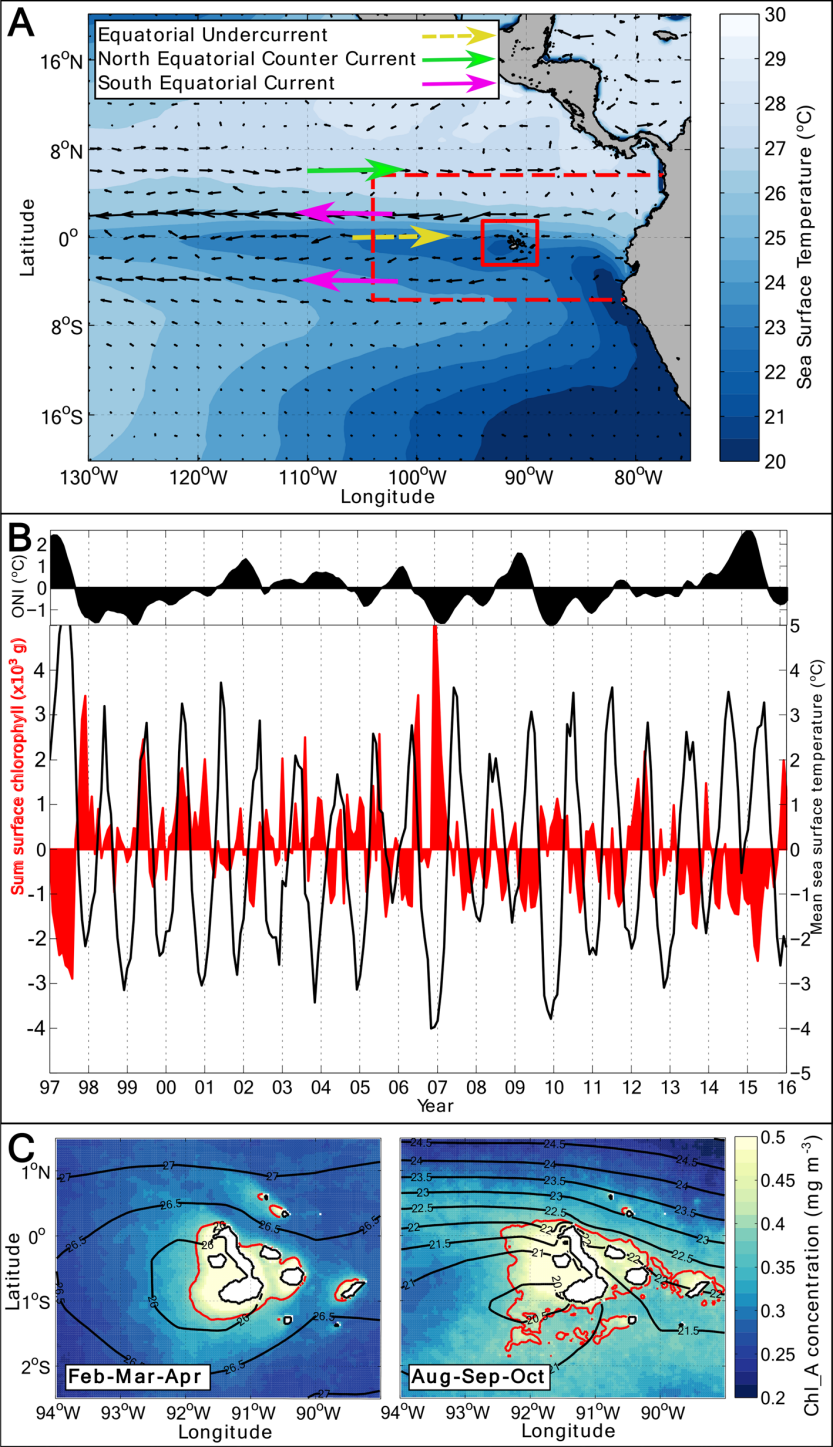
Sea surface temperature, chlorophyll, and near-surface ocean currents around the Galápagos. (**A**) Map of the equatorial Pacific showing major near-surface currents. Sea surface temperature (SST) is the annual mean for 2010 from ERA-Interim^13^, and velocity vectors are from 2010 annual-mean AVISO absolute dynamic topography^42^. The model outer boundary is marked as a dashed red line, with the area of analysis indicated as a solid red line. (**B**) Upper frame shows values of the Ocean Niño Index (ONI https://origin.cpc.ncep.noaa.gov/products/analysis_monitoring/ensostuff/ONI_v5.php), where positive values indicate El Niño conditions. Lower frame shows the monthly integral of surface chlorophyll-*a* anomaly from satellite observations^29^ and monthly-mean SST anomaly from ERA-Interim from 1997 to 2016. The anomalies are calculated as the difference from climatological (1997–2016) mean values. The correlation coefficient between mean SST and integrated chlorophyll-*a* is − 0.4. (**C**) Climatological mean sea surface chlorophyll-*a* concentration for February–April, when SST is warmest, and for August–October, when SST is coldest in the analysis region. Mean SST contours are shown in black, with the 0.4 mg m^−3^ chlorophyll-*a* concentration contour in red.

The Galápagos archipelago, rising from the eastern equatorial Pacific Ocean some 900 km off the South American mainland, hosts an iconic and globally significant biological hotspot. The islands are renowned for their unique wealth of endemic species, which inspired Charles Darwin’s theory of evolution and today underpins one of the largest UNESCO World Heritage Sites and Marine Reserves on Earth. The waters surrounding the Galápagos archipelago (Fig. 1a) are characterized by a plume of high phytoplankton biomass, which forms the base trophic level of the islands’ exceptionally rich and distinctive ecosystem^1^. Although generally elevated relative to the wider equatorial Pacific, phytoplankton biomass around the islands is subject to strong intermittency on time scales of months to years^2^, and exhibits both a prominent seasonal cycle and an acute susceptibility to regional climate extremes^3^. Establishing the causes of the phytoplankton’s marked seasonality and climate sensitivity is key to assess the resilience of the regional ecosystem against the mounting pressures of contemporary climatic change. This is illustrated by the observed correspondence of the most severe perturbations to the islands’ ecosystem in recent decades with major reductions in phytoplankton levels, coinciding at times with strong El Niño events^3^.

The Galápagos phytoplankton stock is sustained by upwelling^1,4^, manifested, for example, in a systematic association between elevated surface concentration of chlorophyll-*a* (indicative of phytoplankton levels) and decreased sea surface temperature (SST) (indicative of cool, nutrient-rich deep waters reaching the surface^5–7^) around the archipelago in satellite measurements (Fig. 1b,c). Present understanding of the upwelling’s causes, dating back to the 1960s, often attributes a pivotal regulatory role to the sub-surface Equatorial Undercurrent (EUC)^1,6–8^ (Fig. 1a). In this view, waters conveyed eastward by the EUC at depths of up to 100 m are thought to rise upon colliding with the islands, such that fluctuations in upwelling are presumed to result from remotely-forced or stochastic changes in the current’s intensity and path. However, observations of the EUC’s impingement on the archipelago are scarce^9,10^, while evidence of the upwelling’s responsiveness to fast-evolving, transitory phenomena such as tropical instability waves^5,11^ and, possibly, wind forcing^12^ suggests that other, more geographically focussed processes may also be at play.

## Results

### Wind control of Galápagos upwelling

**Figure 2:**
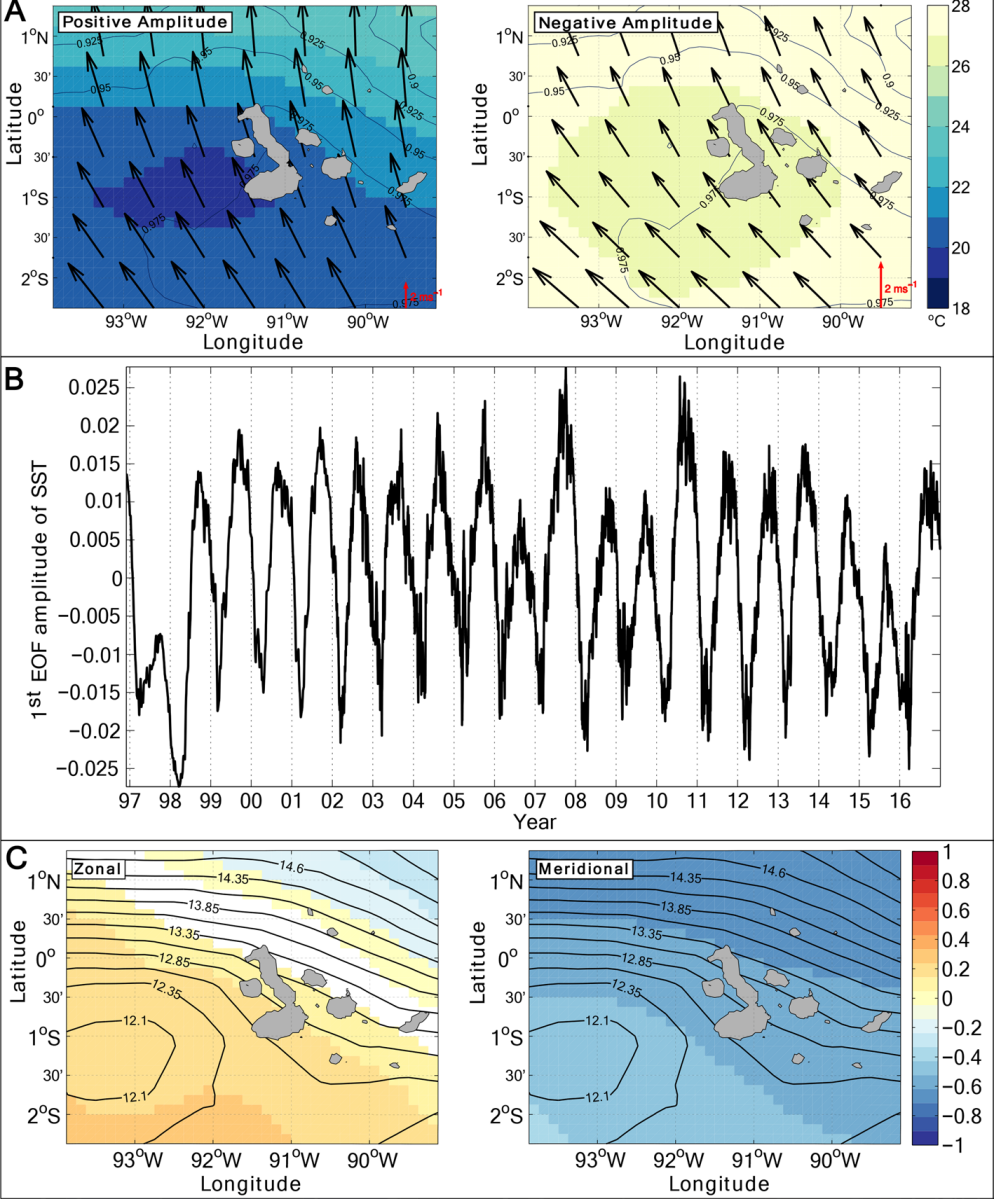
Patterns and correlations of sea surface temperature and wind around the Galápagos. (**A**) Mean sea surface temperature (SST) for days with positive and negative amplitudes of the first empirical orthogonal function (EOF) of ERA-Interim reanalysis SST^13^. Black contours show the spatial correlation between SST and first EOF of SST. Black arrows indicate mean wind velocity vectors for each period. (**B**) Amplitude of the first EOF of SST. (**C**) Maps of the correlation between the first EOF of SST and the zonal and meridional wind stresses. Areas of no colour indicate less than 99% confidence in the correlation (p > 0.01). Black contours show SST for the maximum amplitude of the first EOF (5 October 2007).

To identify these processes, we first consider the temporal variability of SST around the archipelago, and its relationship to wind stress, in an observation-based atmospheric reanalysis^13^ (see “Ocean colour and sea surface temperature processing” in “Methods”). Regional SST regularly exhibits a spatial pattern with a coherent pool of relatively cool waters to the west of the islands between 2°S and the equator, indicating that upwelling predominantly occurs in that area (Fig. 2a). The pattern’s amplitude varies substantially in time, most glaringly on seasonal time scales (Fig. 2b). The pool of cool SST is most pronounced during the dry Garúa season (May to December) and comparatively muted during the hot, wet season (December to May), reflecting an enhancement of upwelling in the former period relative to the latter^6,12^. Cross-correlation of the amplitude of the SST pattern (specifically, the first principal component of the regional SST, which captures the amplitude of the pattern; see “Empirical orthogonal function analysis” in “Methods”) with the zonal and meridional components of the wind stress reveals a significant relationship between SST in the upwelling-prone area to the west of the archipelago and the strength of meridional winds (Fig. 2c). While correlation does not necessarily imply causality, this suggests that local wind forcing might exert an important control on the intensity of Galápagos upwelling.

### Mechanism of wind-driven upwelling

The physical plausibility of such a control, and the dynamics underpinning it, are assessed with a numerical model of the ocean circulation of the eastern equatorial Pacific. The model has sufficiently fine horizontal resolution (4 km) to resolve ocean dynamics down to the submesoscale^14,15^; represents unresolved boundary layer physics via a widely successful parameterization^16^; is forced with atmospheric reanalysis fields at the surface and relaxed to annual-mean hydrographic and velocity fields at the northern, western and southern boundaries, so that the modelled circulation attains a statistically stationary state; and is analysed over a 1-year period (August 2010–July 2011) that is characterised by transitory El Niño Southern Oscillation (ENSO) conditions and sufficiently long to capture the bulk of the range of observed regional SST values (Fig. 1b). The model reproduces the main surface features of the regional circulation and its temporal variability, including: the mean intensity and position of the westward South Equatorial Current (SEC); and the spatial structure, temperature signature and temporal evolution of the pool of cool SST. The model also captures the measured mean intensity and position of the EUC^9,17^ as well as this current’s bifurcation on colliding with the islands^9,10^, albeit the model omits the observed seasonality of the EUC flow^9,17^ by design. See “The MITgcm model” in “Methods” for a full description of the model design and validation. Most significantly, the model replicates the temporal variability in SST to the west of the islands and its relationship to meridional wind stress documented by observations (Figs. 2c and Fig. S3b), endorsing the notion that local atmospheric forcing is the upwelling’s key driver. Thus, in reproducing the measured evolution of SST with time-invariable lateral boundary conditions, our model suggests that temporal changes in the EUC—the traditionally-favoured upwelling-forcing factor—play a lesser role in modulating upwelling (see “The MITgcm model” and “Model float release experiments” in “Methods”). This indicative result should be corroborated in the future with submesoscale-permitting simulations including a seasonally-variable EUC.

**Figure 3:**
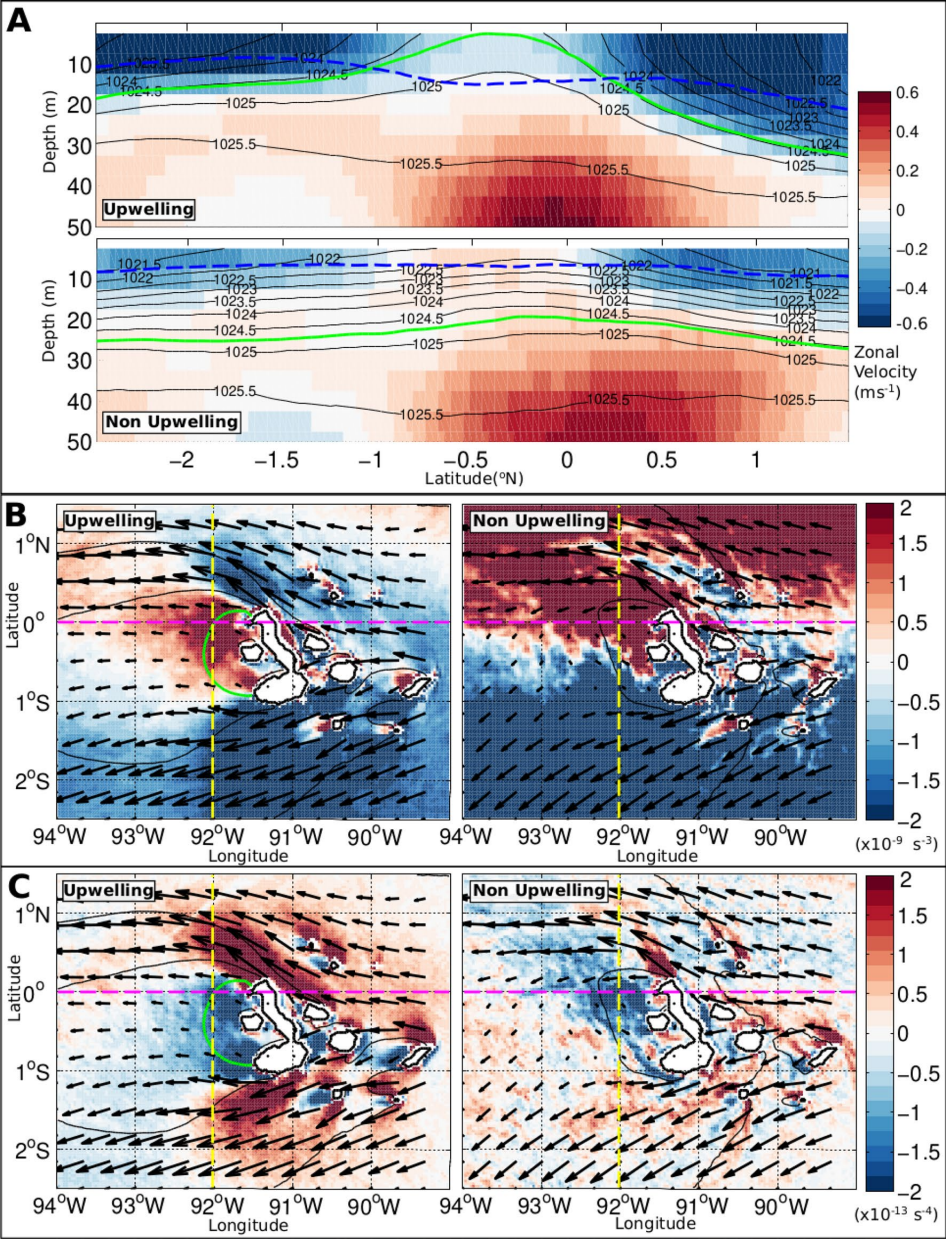
Model stratification and potential vorticity during upwelling and non-upwelling periods. (**A**) A section along 92ºW of mean zonal velocity, with density contours (black), mixed layer depth (blue), and 20ºC isotherm (green) means for periods of upwelling and non-upwelling conditions. (**B**) Full potential vorticity (*Q*, s^−3^) averages for upwelling and non-upwelling periods, along with period-mean surface velocity vectors in black, where water speeds are in the range 0.01–0.83 m s^−1^ for upwelling periods and 0.01–0.73 m s^−1^ for non-upwelling periods. (**C**) *Q* flux vector (${J}_{z}^{F}$, s^−4^) and mean surface velocity vectors in black for upwelling and non-upwelling periods. The location of the density section shown in panel (**A**) is marked as a yellow dashed line, and the equator as a magenta dashed line.

**Figure 4:**
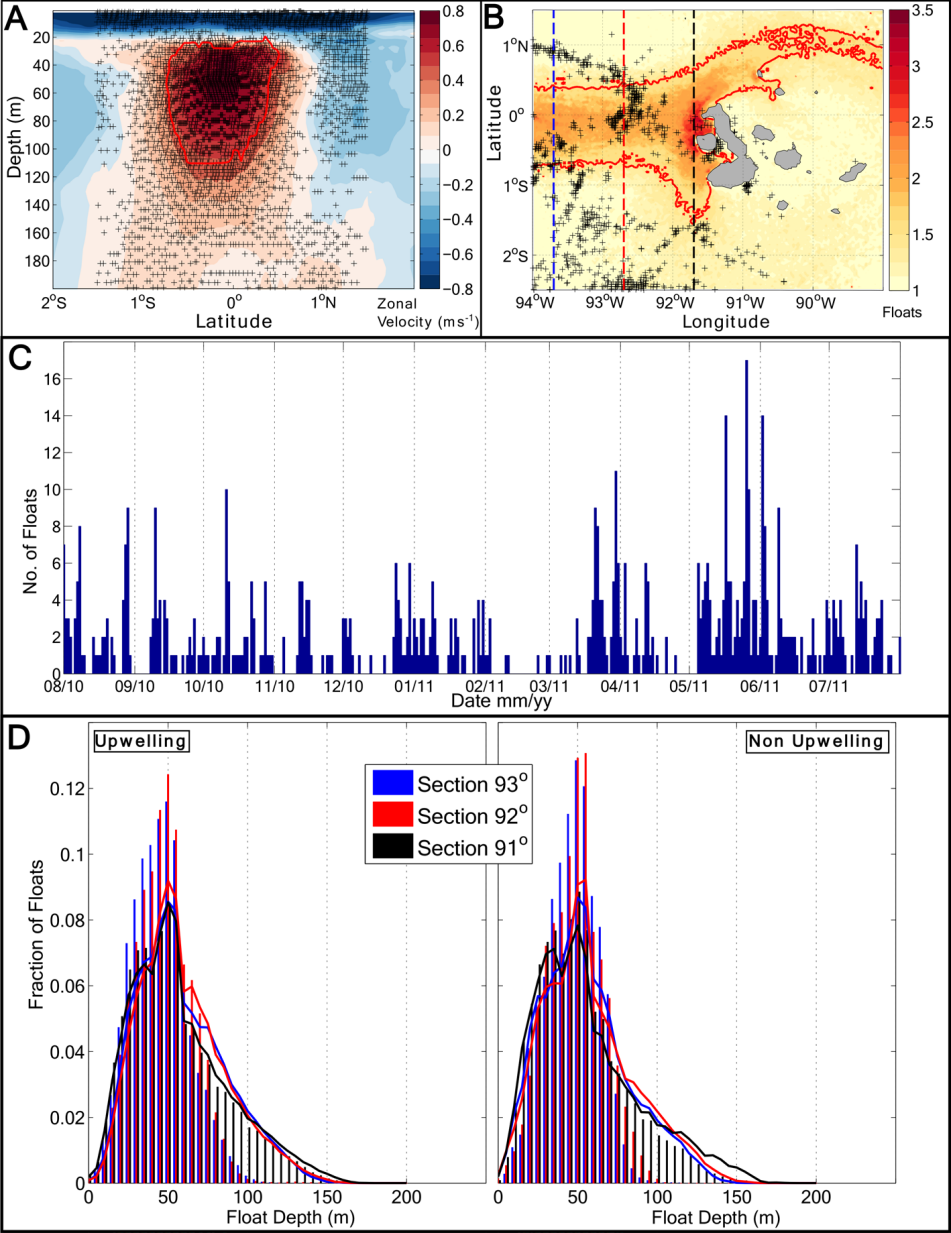
Float depth and positions from the model float release experiments. (**A**) Float deployment positions, marked with black crosses (only every fifth float shown for clarity), and zonal velocity for a typical float deployment. The red contour indicates floats identified as being in the Equatorial Undercurrent (EUC) core. (**B**) Mean per-release float density for all floats passing through the analysis region. Black crosses indicate the positions at which floats enter the model mixed layer. The red contour denotes a mean float density of 1.5 per model grid cell. Coloured dashed lines indicate the positions of the sections referred to in panel (**D**). (**C**) Number of floats entering the model mixed layer for each day of the simulation from all float releases. (**D**) Distribution of depths of floats passing through the analysis region measured at three sections, at 93.7ºW (blue), 92.7°W (red) and 91.7°W (black), for upwelling and non-upwelling periods. Bar plots are for floats identified as being in the core of the EUC, and lines for all floats.

An overview of the mechanism enabling wind-driven upwelling in the model is provided by Fig. 3a. Periods of strong upwelling to the west of the islands (identified by an intensified pool of cool SST) are associated with a pronounced up-doming of isopycnals and reduced upper-ocean stratification around 0.5°S, and with a deeper mixed layer everywhere to the north of 1.5°S. This contrasts with the generalized, strongly stratified conditions and shallow mixed layer occurring at times of no upwelling. The features seen during strong upwelling periods indicate that the wind forcing of upwelling entails a destruction of upper-ocean stratification, which preconditions the mixed layer for efficient deepening via night-time convection^18^. This ‘top-down’ driving of upwelling is also apparent in the trajectories of virtual floats released within the modelled EUC (Fig. 4; see “Model float release experiments” in “Methods”). These exhibit horizontal and vertical pathways that are independent of the intensity of upwelling, except in highly localized areas to the west and northeast of the islands in which a subset of the particles are abruptly entrained into the mixed layer.

### Dynamics of wind-driven upwelling

To elucidate the dynamics behind this entrainment process, the susceptibility of the regional circulation to submesoscale instabilities is assessed by examining the distribution of potential vorticity, *Q*. The definition and procedures for the computation of *Q* and associated variables are described in “Regional ocean dynamics” in “Methods”. A variety of instabilities may develop in a geophysical fluid when *Q* takes the opposite sign to the planetary vorticity^19,20^, which is positive in the Northern Hemisphere and negative in the Southern Hemisphere. The instabilities induce overturning motions (entailing intensified and intermittent vertical velocities) that extract energy from the background flow and expend it in the production of small-scale turbulence, mixing the fluid toward a state of marginal stability. Although our model does not explicitly resolve these overturning motions, the model’s high degree of realism suggests that the generation of the conditions required for instability development is adequately represented, as are the instabilities’ bulk impacts on stratification and mixing (via the boundary layer parameterization).

During periods of no upwelling, surface *Q* is characterized by an abrupt transition from negative to positive values in the close proximity of the equator, indicative of stable conditions (Fig. 3b, right panel). In contrast, at times of strong upwelling, there are substantial areas of anomalously-signed *Q* directly to the west of the islands (positive *Q* to the south of the equator) and to the northwest of the islands (negative *Q* to the north of the equator), where the above-mentioned destruction of upper-ocean stratification and mixed layer deepening are most pronounced (Fig. 3b, left panel). This suggests that the wind regulation of Galápagos upwelling is exerted through the development of submesoscale instabilities in these areas. The role of wind forcing in instability generation is confirmed by examining the rate of frictional (i.e. wind-induced) destruction of *Q* at the ocean surface, denoted ${J}_{z}^{F}$ (Fig. 3c), where a positive ${J}_{z}^{F}$ indicates a destruction of positive *Q* or, equivalently, a production of negative *Q*. During periods of strong upwelling (Fig. 3c, left panel), vigorous production of anomalously-signed* Q* occurs directly to the west of the islands (negative ${J}_{z}^{F}$) and to the northwest of the islands (positive ${J}_{z}^{F}$), precisely in the areas exhibiting unstable conditions in surface *Q* (Fig. 3b, left panel). In turn, frictional destruction of *Q* is comparatively weak at times of no upwelling (Fig. 3c, right panel).

Submesoscale instabilities are respectively termed gravitational, centrifugal or symmetric if the overturning motions extract energy from the vertical stratification, the lateral shear, or the vertical shear and lateral stratification of the background flow^19,20^. The nature of the instabilities underpinning regional upwelling is evaluated by decomposing *Q* into components associated with vertical stratification and lateral shear (*q*_*z*_) and with vertical shear and lateral stratification (*q*_*h*_) (see “Regional ocean dynamics” in “Methods”). The relative magnitudes of these components are examined to the west of the islands, where the sign of surface *Q* is favourable to instability development, at times of strong upwelling, in order to identify the dominant instability type.

**Figure 5:**
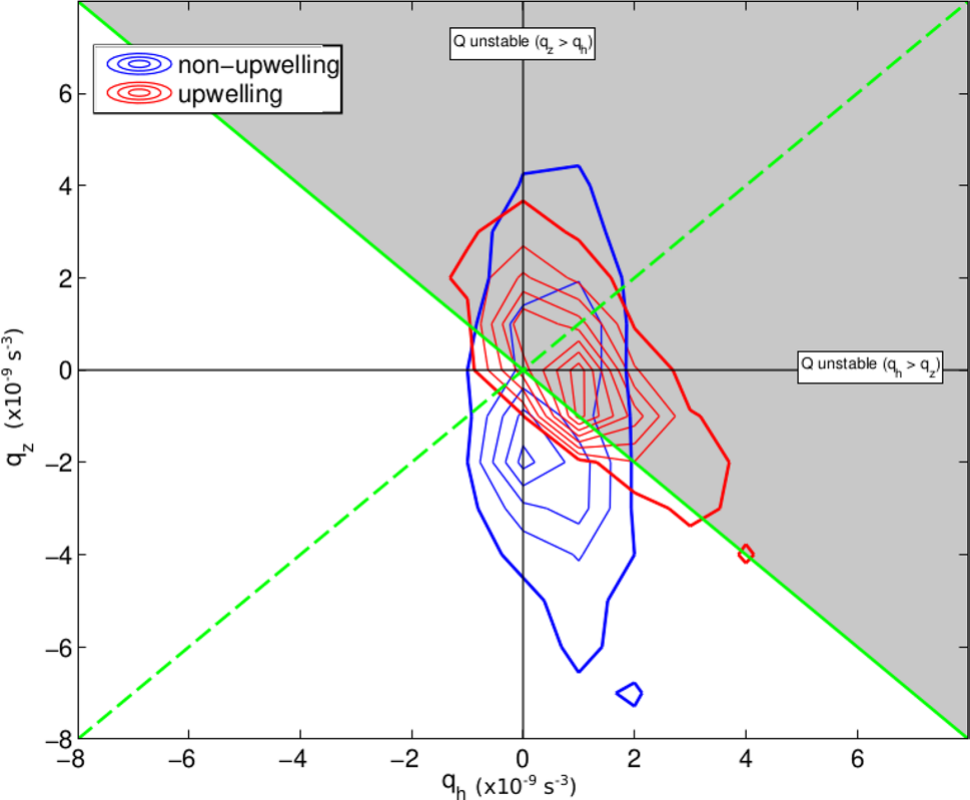
Comparison of horizontal and vertical potential vorticity during upwelling and non-upwelling periods. A 2d-histogram of *q*_*h*_ and *q*_*z*_ (s^−3^) for upwelling (red) and non-upwelling (blue) periods for the area between 0–1°S, 93–91°W. 1% of values contour is indicated using a thicker line. *Q* = 0 (i.e. *q*_*h*_ + *q*_*z*_ = 0) is marked as a thick green line, and *q*_*h*_ = *q*_*z*_ is indicated as a dashed green line. The area of parameter space favourable to the development of submesoscale instabilities is shaded grey.

**Figure 6:**
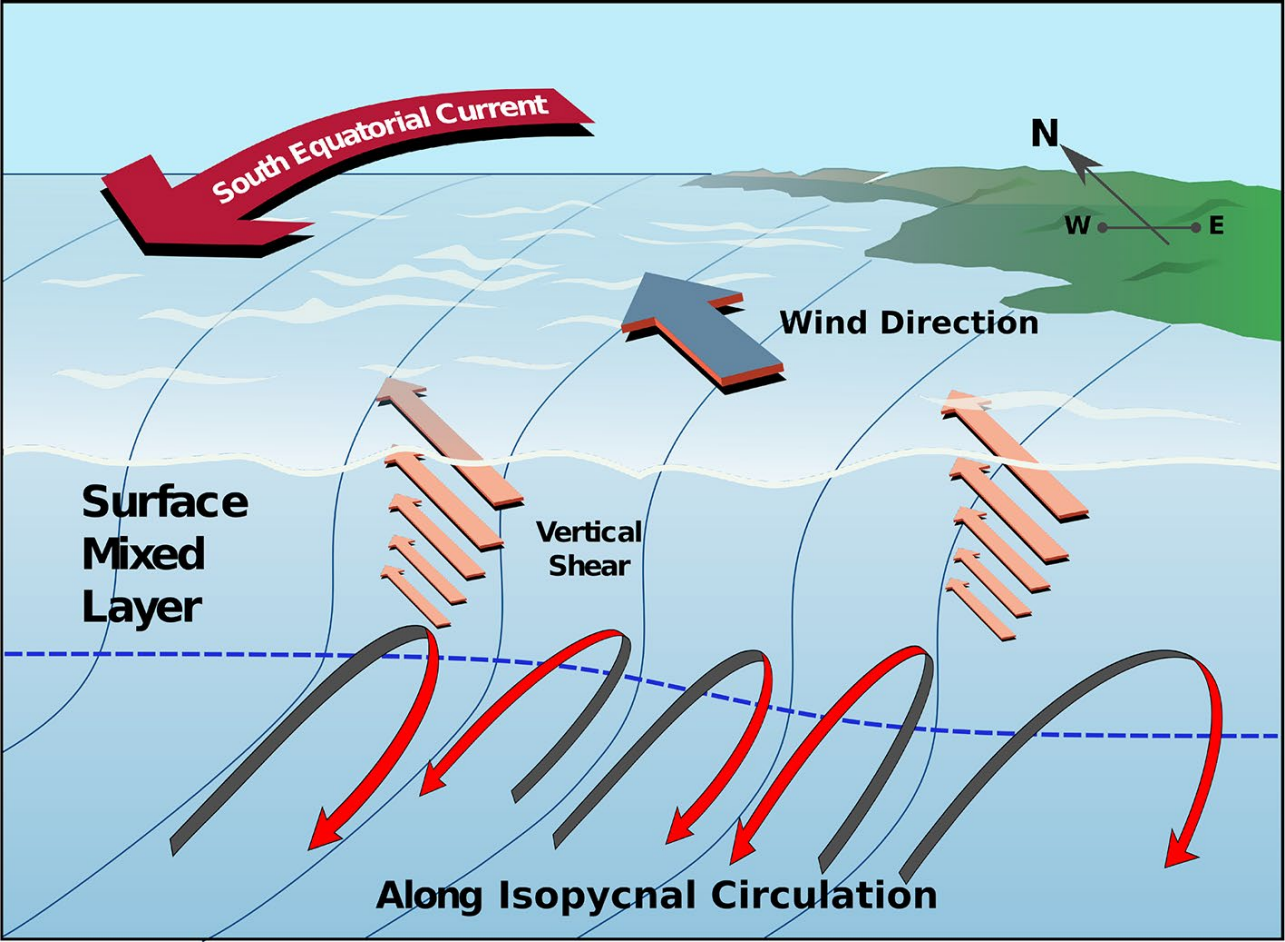
Schematic of the mechanism of wind forcing of Galápagos upwelling. Meridional wind-driven shear in the upper ocean is directed along density fronts near the islands’ western margin, set up by the archipelago’s blocking of the westward-flowing South Equatorial Current. The interaction of the winds with the fronts results in a deepening of the surface mixed layer and the development of (submesoscale) symmetric instability, which induces re-stratifying overturning flows along the sloped isopycnals. The localised mixed layer deepening and along-isopycnal flows promote upwelling of deep waters toward the surface.

The magnitude of *q*_*h*_ is found to regularly exceed that of *q*_*z*_ (Fig. 5), indicating a local prevalence of symmetric instability in inducing upwelling. Symmetric instability develops in this area as northward winds generate meridional vertical shear within the upper ocean, leading to a reduction in vertical stratification (Fig. 3a). The vertical shear combines with an eastward density gradient, permanently present to the west of the archipelago (Fig. 3b,c), to set up a flow regime for which zonally-oriented, overturning motions along density surfaces may grow rapidly^20,21^. These motions act to partially restore vertical stratification by displacing cool interior waters from the west upward and toward the islands’ western margin, and warmer surface waters from the western margin downward and westward, ultimately resulting in enhanced interior-surface exchange and upwelling into the locally deepened mixed layer (Fig. 6).

## Discussion

Our model shows that the wind regulation of Galápagos upwelling is effected via submesoscale instabilities whose generation is contingent not only on forcing by northward winds, but also on the occurrence of substantial lateral density gradients (i.e. fronts) near the ocean surface. Thus, it is the enduring presence of upper-ocean density fronts around the western flank of the archipelago that enables wind forcing to preferentially trigger the instabilities in that area, relative to a wider region of the eastern Pacific forced by similar winds^22^. To assess the physical underpinning of the persistent lateral density gradients to the west of the islands, we conduct additional model simulations in which we alter the archipelago’s position and/or geometry, as well as aspects of the wind forcing (see Suppl. Info. and Figs. S4 and S5). Analysis of this simulation suite reveals that the density fronts are produced by the islands’ blocking of the westward-flowing SEC, which diverts and splits upon impinging on the archipelago’s eastern flank. Subsequently to the split, the SEC’s northern and southern branches accelerate and overshoot the islands’ western rim, inducing upper-ocean horizontal divergence in that area (cf. Fig. 3 and observations in ref.^10^). This in turn results, by continuity, in an adiabatic uplifting of interior density surfaces to the west of the islands. As these uplifted waters are denser than surrounding waters at the same depth, a band of elevated lateral density contrast is generated around the uplift area, preconditioning the region for wind-driven upwelling.

While upper-ocean divergence and uplifted density surfaces are a permanent occurrence to the west of the archipelago, their imprint on lateral density gradients in the area can be modulated by the large-scale circulation of the equatorial Pacific. The clearest instance of this modulation takes place during strong El Niño events, and is illustrated here with an additional simulation with atmospheric forcing and lateral boundary conditions corresponding to the El Niño event of 1997/98 (see Suppl. Info. and Fig. S6). At times of pronounced El Niño conditions, a deepening of the thermocline occurs in the eastern equatorial Pacific that results in reduced vertical stratification in the uppermost portion of the water column^9^. As the vertical density gradient decreases, so does the lateral contrast between the waters uplifted to the west of the islands and surrounding waters. Ultimately, as the upper-ocean density fronts weaken, the rate at which the wind generates anomalously-signed *Q* reduces, and the development of submesoscale instabilities (and upwelling) decreases. Thus, the shutdown of upwelling during strong El Niño events stems not from a substantial change in the winds around the archipelago^22^, but from the suppression of the density fronts predisposing the area to wind-driven upwelling. This finding is corroborated by further simulations that combine atmospheric forcing and vertical stratification taken from different (1997/98 vs. 2010/11) periods (see Suppl. Info.).

In conclusion, our results show that Galápagos upwelling is primarily regulated by northward winds blowing on quasi-permanent upper-ocean fronts to the west of the archipelago, and suggest that traditionally highlighted EUC-associated mechanisms^1,6–8^ need not be invoked to explain the observed (upwelling-induced) variability in SST or—more generally—the characteristic local reduction in SST relative to the wider equatorial Pacific^10^. The wind forcing of the fronts, which are generated by the islands’ blocking of the SEC, results in the development of submesoscale instabilities that promote upwelling via entrainment of interior waters into, and deepening of, the mixed layer (Fig. 6). It is this upwelling that forms the basis of the highly productive region along the western border of the Galápagos Marine Reserve, where the eastern Pacific industrial tuna fleet concentrates its fishing activity^23,24^. Further, the reproductive success of many of the endemic species inhabiting the islands, such as the Galápagos fur seal, Galápagos penguin and flightless cormorant, is highly dependent upon this upwelling^7,25^. With the eastern equatorial Pacific projected to experience increasingly frequent and severe climate extremes in coming decades^26,27^, assessing the ecological resilience of the Galápagos ecosystem stands out as a pressing and formidable challenge^28^. Our findings indicate that an adequate representation of the localized atmosphere–ocean interactions documented here will be critical to the reliability of such assessments and, arguably, the effectiveness of the climate mitigation strategies issued on their basis.

## Methods

### Ocean colour and sea surface temperature processing

A time series of sea surface chlorophyll-*a* concentration in the eastern equatorial Pacific was constructed from monthly-mean satellite observations of ocean colour between October 1997 and December 2016^29^. Gaps in the raw ocean colour measurements were patched, first with the median of a 7 × 7 pixel area centred on the missing-data pixel, and subsequently for any remaining gaps with an 11 × 11 pixel median^2^. Monthly and daily mean time series of sea surface temperature (SST) in the same region were constructed from ERA-Interim^13^ 6-hourly mean SST values between October 1997 and December 2016. The ERA-Interim SST data set is primarily based on satellite observations, and has a horizontal resolution of 1/8° that broadly captures the effects of the Galápagos archipelago.

### Empirical orthogonal function analysis

An empirical orthogonal function (EOF) analysis^30^ was performed on daily-mean ERA-Interim SST data between January 1997 and December 2016, for a region centred on the Galápagos Islands between 2.5°S to 1.5°N and 94 to 89°W (Fig. 1a). Confidence levels (p-values) were calculated in all cases using effective degrees of freedom, allowing for variable autocorrelation. Correlations with less than 99% confidence (p > 0.01) were disregarded. The analysis indicates that as much as ∼ 94% of the total variance of SST is explained by the first EOF, which correlates highly with SST throughout the analysis domain (r = 0.88 to 0.99 with 99% confidence (p < 0.01)). The spatial pattern of this first EOF describes a coherent pool of relatively cool SST to the west of the islands between 2°S and the equator (Fig. 1c), and indicates that upwelling predominantly occurs in that area^5–7^. Note that ERA-Interim air-sea heat fluxes in our analysis region, which are relatively time-invariable, exert only a minor influence on the temporal evolution of SST (not shown). The first EOF’s amplitude varies seasonally, attaining minimum values between January and April as SST west of the islands peaks (Fig. 2a) and upwelling is weakest. Cross-correlation of the first EOF’s amplitude with zonal and meridional wind stresses from ERA-Interim reveals the occurrence of a highly significant anticorrelation between SST and meridional wind stress [r = − 0.66 to − 0.39 with 99% confidence (p < 0.01)] (Fig. 2c, right panel). In contrast, there is a more modest correlation between SST and zonal wind stress [r = − 0.32 to 0.22, significant with 99% confidence (p < 0.01)] (Fig. 2c, left panel). This suggests that there might be a physical link between variations in meridional wind forcing and in SST and, by extension, in the intensity of upwelling. We hereafter use the first EOF’s amplitude as a metric of upwelling intensity, and define amplitudes in excess of -0.04 to correspond to upwelling periods. Our results are insensitive to the precise amplitude chosen for this definition.

### The MITgcm model

The model was constructed using MITgcm^31^ with bathymetry from the General Bathymetric Chart of the Oceans (GEBCO_14)^32^. The model grid’s horizontal resolution was a constant 4 km (0.03334°), with 840 grid points in *x*, 400 in *y*, and a grid origin at 6.667°S, 105°W. The vertical grid comprised 75 depth levels, with vertical resolution varying with depth from 5 m in the uppermost 50 m, 9.8 m down to 164 m depth, 13.7 m down to 315 m depth, to a maximum cell height of 556 m below 3000 m. The model was run in quasi-hydrostatic mode^33^, which incorporates horizontal Coriolis terms in the primitive momentum equations and a non-linear free surface. Horizontal viscosity was parameterised as a biharmonic operator, using a combination of the adaptive Smagorinsky^34^ and Leith^35^ schemes with scaling coefficients of 0.9 and 1.85, respectively. Horizontal diffusion was parameterised with a combined constant biharmonic diffusivity of 10^8^ m^4^ s^−2^. Vertical mixing was represented using the K-profile (KPP) scheme^16^.

In order to focus on the effects of local forcing on Galápagos upwelling, the model was constructed with constant prescribed boundary conditions at its three open boundaries (north, south and west), using constant boundary forcing for temperature, salinity and velocity fields and a 30 grid cell-thick sponge layer. Initial and boundary conditions were taken from the ORCA0083-N01 33-year run of the 1/12 (0.083°) NEMO model forced using the DRAKKAR Forcing Set (https://www.nemo-ocean.eu/)—a simulation that has been carefully validated with observations, and shown to adequately represent the key circulation features of the equatorial Pacific (see ref.^36^, and references therein). NEMO model 2010 annual-mean temperature, salinity and horizontal velocity fields were interpolated onto our model grid. The model was initially run for 5 years without surface forcing so as to bring the ocean into a statistically equilibrated state. Subsequently, the model was run with surface forcing fields (wind stress, radiation, evaporation and precipitation) taken from ERA-Interim at 6-hourly temporal resolution. No unphysical relaxation to observed sea surface properties was applied, in order to avoid obscuring the physical relationship between atmospheric forcing and such properties. Simulations began on 1 January 2010 and concluded on 31 December 2011. Analysis was conducted from 1 August 2010.

The model has a realistic representation of the main features of the regional surface circulation and its variability, such as the mean intensity and position of the westward-flowing South Equatorial Current (SEC) and its split into two branches upon impinging on the Galápagos Islands (Fig. S1a). The spatial patterns of surface velocity variability measured by surface drifters are also broadly captured by the model (Fig. S1b). The mean intensity and position of the Equatorial Undercurrent (EUC) are adequately reproduced by the model too (Fig. S2). Although the EUC’s volume transport is slightly lower than daily-mean transport values diagnosed from the Tropical Atmosphere–Ocean (TAO) array^9,17^, the modelled transport commonly lies within one standard deviation of the observed transport (Fig. S2a). Similarly, the depth of the EUC’s velocity core in the model is within the range of the daily-mean observed values (Fig. S2b), as is the depth of the 20 °C isotherm within the EUC (Fig. S2c). Note, however, that the model does not capture the marked seasonal variability in the EUC’s intensity and depth revealed by the observations (Fig. S2b–c). This is a design feature of the model, chosen to discriminate between the roles of local and remote (e.g., EUC-associated) drivers in modulating upwelling to the west of the islands. The EUC’s observed shoaling as it approaches the archipelago and its subsequent bifurcation around the islands^9,10^ are also realistically represented by the model (Fig. 4b).

Absent a relaxation to observed surface conditions, the model’s upper-ocean temperature drifted away from those conditions. However, the resulting SST bias is both moderate in magnitude (largely smaller than ± 2 °C) and nearly time-invariable (standard deviation of 0.4 °C) for our analysis period, such that our assessment of the links between temporal variations in atmospheric forcing and in SST is unaffected by the bias. Most importantly, the model realistically reproduces the horizontal structure, vertical stratification, temperature anomaly signature and temporal evolution of the pool of cool waters to the west of the archipelago, where upwelling is focussed. This is shown by repeating the EOF analysis previously conducted on ERA-Interim SST (Fig. 2) with the model’s SST between August 2010 and July 2011 (Fig. S3). As in the case of the reanalysis fields, a dominant first EOF is found that accounts for the bulk (in this case, ∼ 80%) of the SST variance, correlates highly with SST throughout the analysis domain [r = 0.59 to 0.92, significant with 99% confidence (p < 0.01)], and describes the coherent pool of cool SST in the upwelling-prone region to the west of the islands (Fig. S3b). The first EOF’s amplitude varies seasonally, with minimum values (indicating warm SST and minimal upwelling) between January and April as in the case of the ERA-Interim SST analysis (Fig S3a). Finally, the model represents the highly significant anticorrelation between SST and meridional wind stress [r = − 0.76 to − 0.56 with 99% confidence (p < 0.01)] documented in the reanalysis data (Fig. 2c). Correlations between SST and zonal wind stress were not significant for either the model or reanalysis data due to the shorter (1 year) duration of the time series analysed (Fig. S3b). All in all, these favorable comparisons indicate that: (i) the temporal evolution of upwelling off the Galápagos Islands can be accounted for by local atmospheric forcing, without the need to appeal to changes in the EUC inflowing the region from the west; and (ii) the model is able to replicate the oceanic processes enabling wind-regulated upwelling.

### Model float release experiments

In order to develop the distinction between the roles of the EUC and wind forcing in regulating Galápagos upwelling, a series of float release experiments were performed in the model using TRACMASS, a particle-tracking application of established skill^37^. Batches of floats were released every 10 days for the duration of the simulation, starting on 5 April 2010. Each batch comprised between 18,000 and 28,000 floats, distributed over the 1.5°S–1.5°N latitude range and the 0–200 m depth range (spanning the EUC core) at 101°W according to the instantaneous zonal transport, with one float per mSv (Fig. 4a), where 1 mSv = 10^3^ m^3^ s^−1^. All floats entering our area of interest (2.5°S–1.5°N and 94–89°W, Fig. 1a) during the analysis period were subsequently considered.

The stream of floats encounters the archipelago at the equator and splits into two branches (cf. observations in ref.^10^), one of which passes to the north of the islands (Fig. 4b). There is no obvious spatial pattern to the floats’ mixed layer entry, and no detectable dependence of the rate at which floats enter the mixed layer on the occurrence or absence of upwelling (cf. Fig. 4b,c). Inspection of the evolving vertical distribution of floats at three meridional sections (at 93.7°W, 92.7°W and 91.7°W; Fig. 4b) reveals no significant change in the mean depth of the float swarm as it approaches the islands (Fig. 4d). The mean depth of floats is 55 ± 27 m, 57 ± 29 m and 56 ± 3 3 m at each of the three sections, respectively, during upwelling periods; the equivalent values when upwelling is absent are 58 ± 27 m, 59 ± 27 m and 58 ± 30 m. Thus, our float release experiments indicate that there is no relationship between the pathways of the EUC and upwelling intensity in the model. Upwelling of floats into the surface mixed layer is instead driven ‘from the top down’, as the floats are captured by a deepening mixed layer—where this deepening is governed by wind forcing via the processes discussed in the main text.

### Regional ocean dynamics

Our assessment of the regional ocean dynamics regulating Galápagos upwelling is founded on the calculation of potential vorticity (*Q*), which is a conserved property of water parcels in the absence of friction and diabatic processes. Generally, the spatial distribution of potential vorticity governs the behaviour of a fluid on time scales relevant to planetary geophysical flows. *Q* can be expressed as:

$${Q}=\left(\mathbf{f}+\nabla \times \mathbf{u}\right).\nabla {b}$$

where $\mathbf{u}=\left(u,v,w\right)$ is the (3-d) velocity vector, **f** the Coriolis vector, *b* is buoyancy ($b =-\mathrm{g}\frac{\uprho -{\uprho }_{0}}{{\uprho }_{0}}$), *g* acceleration due to gravity, *ρ* density and *ρ*_0_ a reference density. Expanding this expression and decomposing it into vertical and horizontal terms (*Q* = *q*_*z*_ + *q*_*h*_) gives:

$${q}_{z}=\left({f}_{z}+\frac{\partial v}{\partial x}-\frac{\partial u}{\partial y}\right).\frac{\partial b}{\partial z}$$

and

$${q}_{h}=\left(\frac{\partial w}{\partial y}-\frac{\partial v}{\partial z}\right).\frac{\partial b}{\partial x}+\left({f}_{y}+\frac{\partial u}{\partial z}-\frac{\partial w}{\partial x}\right).\frac{\partial b}{\partial y}$$

where $\frac{\partial b}{\partial z}$ is the squared buoyancy frequency *N*^2^, and *f*_*z*_ = 2Ω sin *φ* and *f*_*y*_ = 2Ω cos *φ* are the vertical and horizontal components of the Coriolis vector, respectively (Ω is the Earth’s angular velocity and *φ* is latitude)^19^. Neglecting vertical velocity terms^38^, the definition of *q*_*h*_ may be simplified to:

$${q}_{h}=-\frac{\partial v}{\partial z}.\frac{\partial b}{\partial x}+\left({f}_{y}+\frac{\partial u}{\partial z}\right).\frac{\partial b}{\partial y}$$

This set of expressions provides the basis for our diagnostics of the drivers and dynamical controls of upwelling, which are outlined next.

In order to illustrate the way in which wind forcing generates the anomalously-signed *Q* promoting submesoscale instabilities and upwelling, we consider the potential vorticity conservation equation^39,40^,

$$\frac{\partial Q}{\partial t}=-\nabla .\mathbf{J}$$

$$\mathbf{J}=Q\mathbf{u}+\nabla b\times \mathbf{F}-D\left(\mathbf{f}+\nabla \times \mathbf{u}\right)$$

which asserts that the temporal evolution of *Q* may be induced by advection (*Q***u**), diabatic forcing ($D=\frac{d b}{d t}=\mathbf{u}.\nabla b+\frac{\partial b}{\partial t}$) or frictional forcing (**F**). The combination of these three contributions defines the potential vorticity flux vector **J**. As advection cannot by itself reverse the sign of *Q*, and *D* generally counteracts the production of anomalously-signed *Q* in our study region (due to the intense air-to-sea heat fluxes that are prevalent in the equatorial oceans), our analysis focuses on **F**. Specifically, we examine the vertical (upward) frictional flux of potential vorticity through the ocean surface associated with wind forcing, ${J}_{z}^{F}$, which is given by

$${J}_{z}^{F}={\nabla }_{h}\times \mathbf{F}$$

where $\mathbf{F}=\frac{\mathbf{\uptau }}{{\uprho }_{0}{d}_{E}}$ is the frictional force on the ocean due to wind stress **τ**, and *d*_*E*_ the vertical extent of the wind-influenced surface layer^39,40^. Given the proximity to the equator, we take *d*_*E*_ to be the mixed layer depth, diagnosed from the model results using a 0.8 ºC temperature criterion^41^. In our model, northward winds generate a negative ${J}_{z}^{F}$, which in the Southern Hemisphere extracts negative *Q* from the ocean (or, equivalently, inputs positive *Q* to the ocean) and thereby drives the ocean toward instability.

Submesoscale overturning instabilities develop in areas where *f*_*z*_* Q* < 0^19,20^. When this criterion is met, the nature of the instability may be determined as follows. Gravitational instability occurs when *N*^2^ < 0. Centrifugal instability develops when *q*_*z*_ and the absolute vorticity have the opposite sign to *f*_*z*_. Finally, symmetric instability is active when *q*_*z*_ has the same sign as *f*_*z*_, *q*_*h*_ has the opposite sign to *f*_*z*_, and *abs*(*q*_*h*_) > *abs*(*q*_*z*_).

The underpinning of Galápagos upwelling by submesoscale instabilities is indicated by the association of the amplitude of the first EOF of the model’s SST (a metric of the intensity of upwelling; see “Empirical orthogonal function analysis” in “Methods”) and anomalously-signed *Q* in the upwelling-prone area to the west of the islands (Fig. 3c). When the first EOF’s amplitude is large (i.e. upwelling is intense), meridional wind forcing drives surface *Q* toward unstable conditions to the west/northwest of the islands, and the resulting instability leads to a local reduction in upper-ocean stratification and a deepening of the mixed layer (Fig. 3). A detailed examination of the relative contributions of *q*_*z*_ (which amalgamates the terms linked to gravitational and centrifugal instabilities) and *q*_*h*_ (the term linked to symmetric instability) to the anomalously-signed *Q* in the upwelling-prone area demonstrates a prevalence of the *q*_*h*_ term during upwelling periods (Fig. 5). At those times, *q*_*h*_ is dominated by the product of meridional shear and zonal buoyancy gradient (i.e. the $\frac{\partial v}{\partial z}.\frac{\partial b}{\partial x}$ term). Thus, the model indicates that symmetric instability is the key dynamical process mediating the wind forcing of Galápagos upwelling, and suggests that the instability stems from the wind’s generation of large meridional shear across the westward buoyancy gradient to the west of the islands.

## Supplementary Information

Supplementary Information.

## Data Availability

The code for the model used in this research and the data used in the model preparation are all publicly available from the sources cited.
