# Supplementary material for: Galápagos upwelling driven by localized wind–front interactions
Source: Sci Rep. 2021 Jan 14;11:1277. doi: 10.1038/s41598-020-80609-2 (PMC7809033; doi:10.1038/s41598-020-80609-2)
Supplement: Supplementary file 1 — Supplementary Information. [file 41598_2020_80609_MOESM1_ESM.pdf]

Supplementary Information

Galápagos upwelling driven by localized wind – front interactions

Alexander Forryan, Alberto C. Naveira Garabato, Clément Vic, A. J. George Nurser and Alexander R. Hearn

Supplementary Text

Sensitivity of Galápagos upwelling to the islands' geography and geometry

The Galápagos Islands appear to be optimally placed and configured to maximise the upwelling impacts of the climatological winds and surface currents. Our study suggests that the most important factors in determining the strength of the cool water pool to the west of the islands are: (i) the extent to which the SEC is diverted by the archipelago's bathymetry, which influences the strength of the local adiabatic uplifting of isopycnals to the west of the islands; and (ii) the orientation of the resulting westward buoyancy gradient relative to the local wind stress. Given the northward orientation of the climatological mean winds, this buoyancy gradient is only favourable to the development of symmetric instability in the Southern Hemisphere, where the bulk of the islands lie.

In order to qualitatively assess how the geography and geometry of the Galápagos Islands affects the intensity of upwelling, we conducted a series of model experiments where the position and shape of the islands are changed. Of particular note are the 'North' scenario (in which the islands were shifted to lie exclusively within the Northern Hemisphere) and the 'Cylinder' scenario (in which the complex bathymetry of the islands was replaced by a simple cylinder straddling the equator between 0.5ºS and 0.5ºN). Simulations were run using the same configuration as the primary simulation (see *The MITgcm model* in *Methods*), with two exceptions. First, in all supplementary runs only the uppermost 200 m was represented, retaining the same

vertical grid as in the primary simulation. Second, in all supplementary runs the ocean was initialised at rest with a constant stratification and no boundary forcing. Surface forcing is displaced where appropriate, to match the position of the displaced bathymetry.

Both simulations generate a broadly realistic upper-ocean current system and seasonal cycle in SST (Fig. S4a and S5a). However, the area of the cool water pool to the west of the islands is smaller in both the North and Cylinder simulations compared to the primary model run, suggesting that upwelling is reduced in both supplementary simulations. The North simulation exhibits the smallest cool water pool, and thereby weakest upwelling. The density distributions along 92ºW (Fig. S4b and S5b) show the characteristic uplift of isopycnals during upwelling periods, relative to conditions of no upwelling, that is seen in the primary simulation. Surface fields of Q in the supplementary runs also display the same patterns as obtained in the primary simulation, with a wider occurrence of near-zero Q values during upwelling periods than for conditions of no upwelling. However, neither the North nor the Cylinder simulations exhibit the extensive areas of anomalously-signed Q that are present in the primary run to the west of the islands, and that are critical to mediating wind-forced upwelling in that simulation (Fig. S4c and S5c).

All in all, these supplementary model runs highlight that the positioning of the islands in the Southern Hemisphere and their rugged, divergence-inducing bathymetric configuration are important in maximising the response of local upwelling to the prevailing wind forcing.

Shutdown of Galápagos upwelling during El Niño

In order to assess the mechanism behind the widely reported shutdown of Galápagos upwelling during El Niño **[S1, S2]**, a further model simulation was run using the same configuration as the primary simulation but with initial conditions, surface and boundary forcings for 1997/98 – a period with especially strong El Niño conditions. NEMO model annual-mean temperature, salinity and horizontal velocity fields were interpolated onto our model grid. The model was initially run for five years without surface forcing. Subsequently, the model was run with surface forcing fields (wind stress, radiation, evaporation and precipitation) taken from ERA-Interim at 6-hourly temporal resolution. Simulations began on 1 January 1997 and concluded on 31 December 1998. Analysis was conducted from 1 August 1997.

Analysis of the SST fields in this simulation yields qualitatively similar results to those of the primary run (cf. Fig. 3 and S6). A distinct pool of cool water emerges to the west of the islands. However, the SST perturbation associated with this cool water pool is substantially smaller for the 1997/98 simulation than for the primary run, while mean SST is higher across the entire analysis area in 1997/98. These differences manifest that upwelling is strongly suppressed during El Niño. The main causing factor of this upwelling shutdown is the considerably weaker initial upper-ocean stratification of the 1997/98 run (Fig. S6), which results from the large-scale adjustment of the equatorial Pacific pycnocline to El Niño conditions in the NEMO model **[22, 36]**. Reduced vertical stratification translates into weaker, SEC blocking-induced upper-ocean horizontal buoyancy gradients and uplift of isopycnals to the west of the islands. Lack of substantial horizontal buoyancy contrasts implies that local wind forcing (which is little different in 1997/98 relative to other years **[22]**) is inefficient at generating the anomalously-signed Q required for the development of the submesoscale instabilities

underpinning upwelling (Fig. S6). Thus, the shutdown of upwelling during El Niño events stems not from a notable change in the winds around the archipelago, but from the suppression of the density fronts preconditioning the area for wind-driven upwelling.

This conclusion is confirmed with two further simulations (not shown), in which either atmospheric forcing from 1997/98 is combined with stratification from 2010/11 (simulation Mixed-1), or atmospheric forcing from 2010/11 is combined with stratification from 1997/98 (simulation Mixed-2) – following the supplementary run methodology outlined in the section above. Whereas simulation Mixed-1 yields quasi-identical results to those of our control simulation (Fig. 3), simulation Mixed-2 produces the same shutdown of upwelling as the 1997/98 simulation (Fig. S6).

Supplementary References

S1. Firing, E., Lukas, R., Sadler, J. & Wyrtki, K. Equatorial Undercurrent disappears during 1982–1983 El Niño. *Science* 222, 1121–1123. https://doi.org/10.1126/science.222.4628.1121 (1983).

S2. Vargas, F. H., Harrison, S., Rea, S. & Macdonald, D. W. Biological effects of El Niño on the Galápagos penguin. *Biol. Conserv.* 127, 107–114. https://doi.org/10.1016/j.biocon.2005.08.001 (2006).

S3. Laurindo, L. C., Mariano, A. J. & Lumpkin, R. An improved near-surface velocity climatology for the global ocean from drifter observations. *Deep Sea Res. I* 124, 73–92. https://doi.org/10.1016/j.dsr.2017.04.009 (2017).

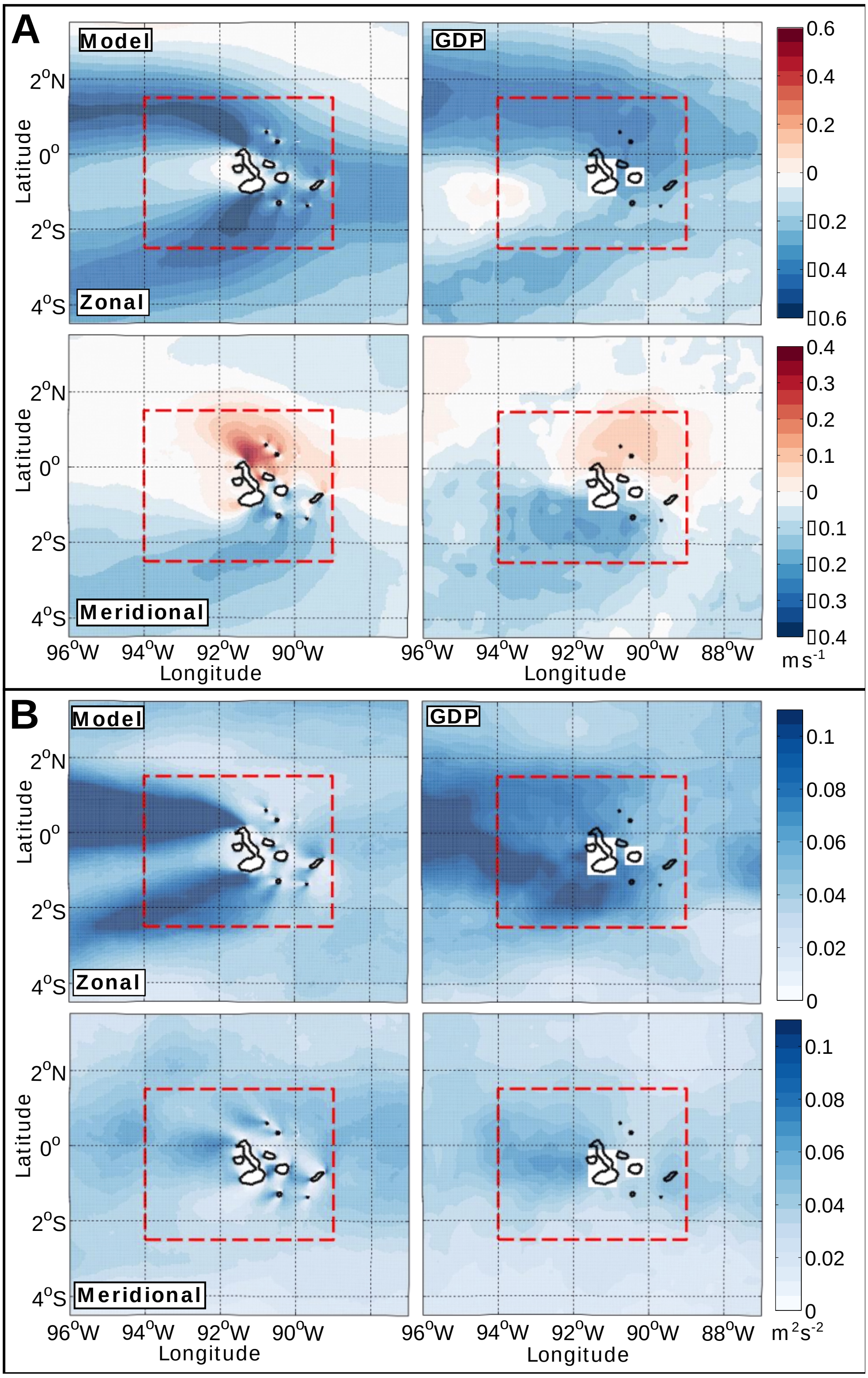

Figure S1 | A comparison between model and observed sea surface currents. (a) Mean model mixed-layer zonal and meridional velocities, and zonal and meridional velocity climatologies from the Global Drifter Program (GDP) **[S4]**. Note the different

velocity scales used. (b) Mean model mixed-layer zonal and meridional velocity variances, and meridional and zonal velocity variance climatologies from the GDP. The analysis area (2.5ºS to 1.5ºN, and 94 to 89ºW, Fig. 1a) is marked by the dashed red line.

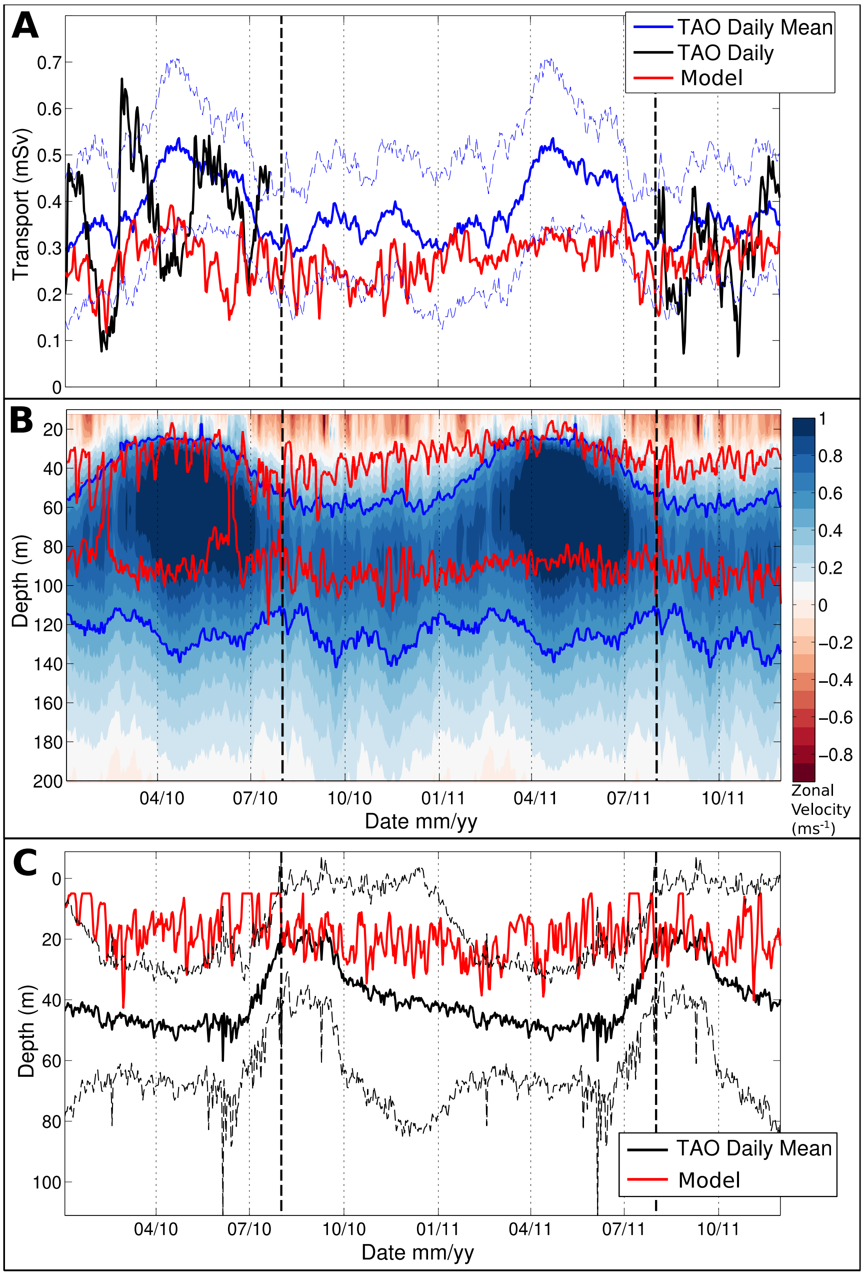

Figure S2 | A comparison between model and TAO mooring array-measured Equatorial Undercurrent (EUC). (a) Model EUC transport (mSv, in red) is compared to daily transport estimated from zonal velocity measured by the Tropical Atmosphere Ocean (TAO) array at 0ºN, 110ºW (https://www.pmel.noaa.gov/gtmba/pmel-theme/pacific-ocean-tao). Daily transport from the TAO array for the period shown is given in black, and climatological-mean (multi-annual) daily transport (±1 standard deviation) is displayed in blue. Vertical dashed lines indicate the start and end of the model analysis period. Transports were

estimated by interpolating the TAO zonal velocity measurements onto the model vertical grid and, for both model and interpolated observations, scaling by the area of the western side of the model grid. (b). Mean daily zonal velocity measured by the TAO array. The blue contour indicates a speed of 0.6 m s^{-1} from the TAO array, and the red contour denotes a speed of 0.6 m s^{-1} in the model. (c) Climatological-mean (multi-annual) depth of the 20ºC isotherm observed by the TAO array at 0ºN, 95ºW (in black, ±1 standard deviation), with the model's representation of that isotherm at the same location shown in red.

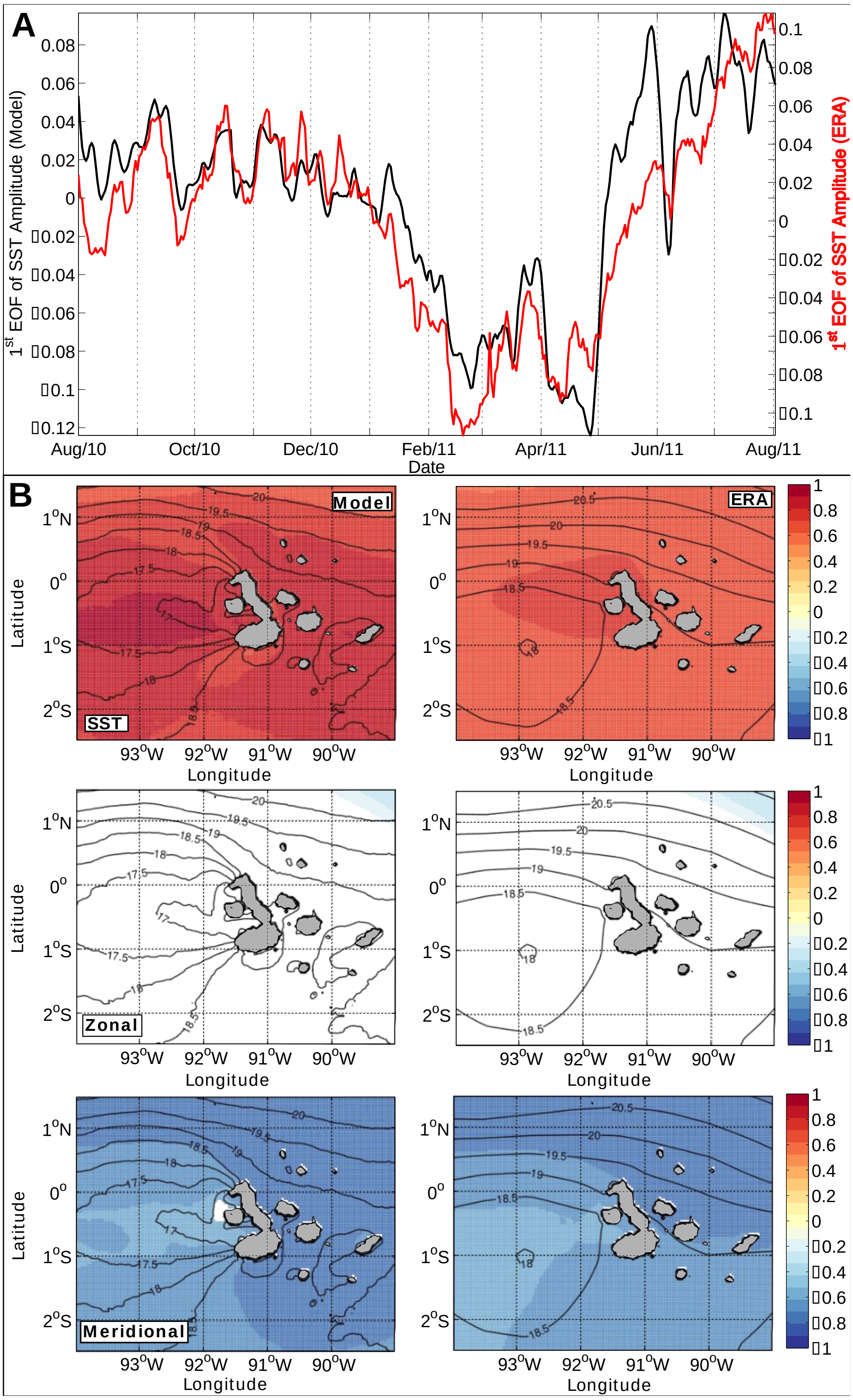

Figure S3 | A comparison between model and ERA-Interim reanalysis sea surface temperature. (a) Amplitude of first empirical orthogonal function (EOF) of sea surface temperature (SST) from model mixed layer temperature (black left-hand axis)

and from ERA-Interim SST (red right-hand axis) for the period August 2010 to July 2011. (b) Maps of the correlation (left column) between first EOF of model mixed layer temperature and model mixed layer temperature (top), zonal wind stress (middle), and meridional wind stress (bottom); and (right column) between first EOF of ERA-Interim SST and ERA-Interim SST (top), zonal wind stress (middle), and meridional wind stress (bottom). Areas of no colour indicate less than 99% confidence in the correlation ($p > 0.01$). Black contours show (left column) model mixed layer temperature or (right column) ERA-Interim SST for the maximum amplitude first EOF during the analysis period (August 2010 to July 2011), on 6 and 31 July for the model and ERA-Interim respectively.

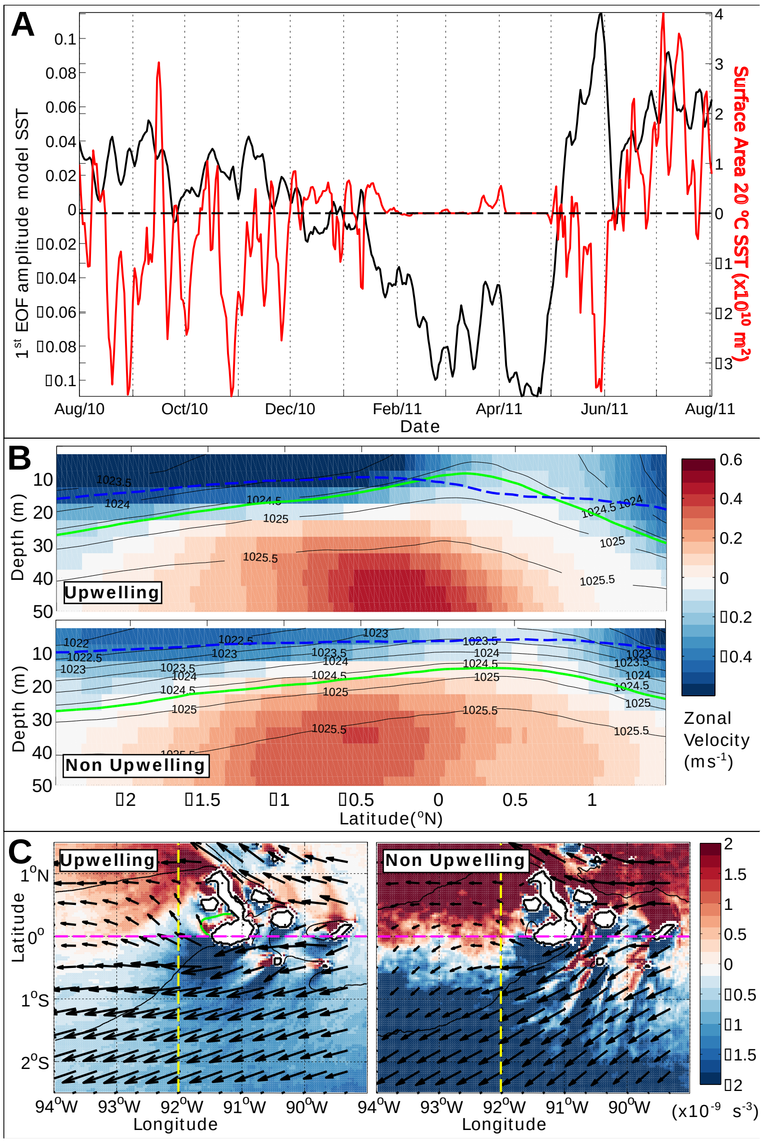

Figure S4 | Patterns of sea surface temperature, stratification, and potential vorticity around the Galápagos for the North simulation. (a) Amplitude of first empirical orthogonal function (EOF) of sea surface temperature (SST) from model mixed-layer temperature for the North simulation (black left-hand axis), and difference in surface area of water colder than 20ºC between the North and primary simulations (red right-hand axis). (b) A section along 92ºW of mean zonal velocity, with density contours (black), mixed layer depth (blue) and 20ºC isotherm (green) means for periods of upwelling and no upwelling from the North simulation. (c) Potential vorticity averages for upwelling and non-upwelling periods from the North simulation. The location of the density section in panel (b) is shown as a yellow dashed line. The equator

is marked as a magenta dashed line. The black vectors indicate period-mean surface velocity, where water speeds are in the range 0.01 - 0.43 m s^{-1} for upwelling periods and 0.01 - 0.82 m s^{-1} non-upwelling periods.

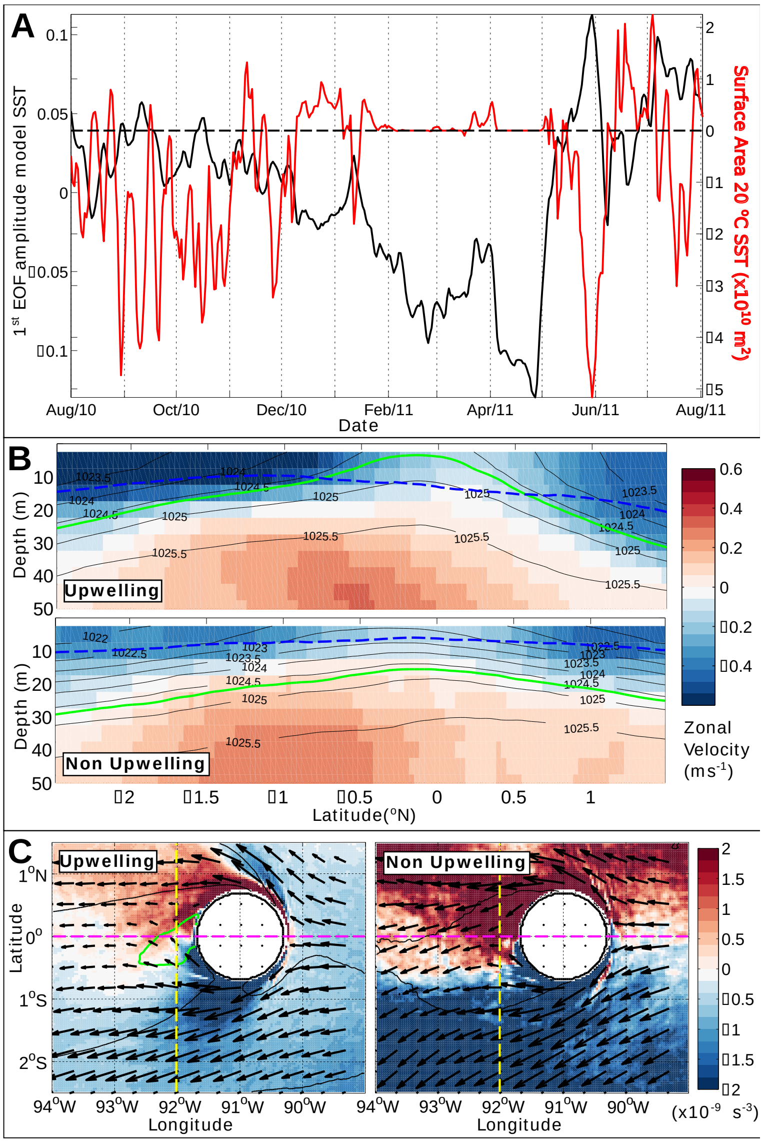

Figure S5 | Patterns of sea surface temperature, stratification, and potential vorticity around the Galápagos for the Cylinder simulation. (a) Amplitude of first empirical orthogonal function (EOF) of sea surface temperature (SST) from model mixed-layer temperature for the Cylinder simulation (black left-hand axis), and difference in surface area of water colder than 20ºC between the Cylinder and primary simulations (red right-hand axis). (b) A section along 92ºW of mean zonal velocity, with density contours (black), mixed layer depth (blue) and 20ºC isotherm (green) means for periods of upwelling and no upwelling from the Cylinder simulation. (c) Potential vorticity averages for upwelling and non-upwelling periods from the Cylinder

simulation. The location of the density section in (b) is shown as a yellow dashed line. The equator is marked as a magenta dashed line. The black vectors indicate period-mean surface velocity where, water speeds are in the range 0.01 - 0.85 m s^{-1} for upwelling periods and 0.01 - 0.64 m s^{-1} non-upwelling periods.

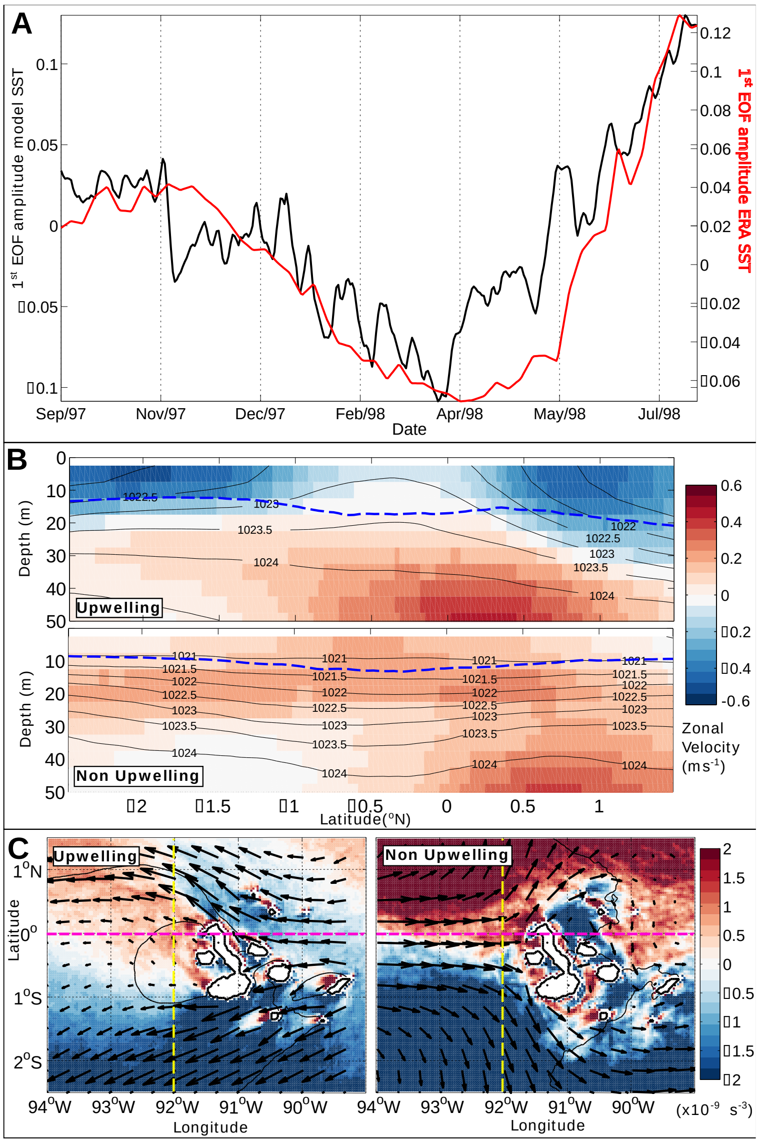

Figure S6 | Patterns of sea surface temperature, stratification, and potential vorticity around the Galápagos for the 1997/98 simulation. (a) Amplitude of first empirical orthogonal function (EOF) of sea surface temperature (SST) from model mixed layer temperature (black left-hand axis) and from ERA-Interim SST (red right-hand axis) for the period September 1997 to August 1998. (b) A section along 92°W of mean zonal velocity, with density contours (black), mixed layer depth (blue) and 20˚C isotherm (green) means for periods of upwelling and no upwelling from the 1997/98 simulation. (c) Potential vorticity averages for upwelling and non-upwelling periods from the 1997/98 simulation. The location of the density section in (b) is shown as a

yellow dashed line. The equator is marked as a magenta dashed line. The black vectors indicate period-mean surface velocity, where water speeds are in the range 0.01 - 0.71 m s^{-1} for upwelling periods and 0.01 - 0.57 m s^{-1} non-upwelling periods.
